# Limb salvage in multiple revision total knee arthroplasty using customised implants: When sleeves and cones are no longer an option

**DOI:** 10.1002/jeo2.70647

**Published:** 2026-01-19

**Authors:** Stefanie Donner, Clemens Gwinner, Henryk Haffer, Carsten Perka, Stephanie Kirschbaum

**Affiliations:** ^1^ Charité – Universitätsmedizin Berlin, Humboldt‐Universität zu Berlin, Center for Musculoskeletal Surgery (CMSC) Berlin Germany

**Keywords:** bone defect, customised, limb salvage, revision total knee arthroplasty, stem fixation

## Abstract

**Purpose:**

Multiple revision total knee arthroplasty (rTKA) remains highly challenging due to severe bone defects, which often render standard implants unsuitable. This study aimed to evaluate the clinical outcomes, survivorship and complication rates of customised knee implants used in aseptic one‐stage rTKA for patients with severe bone defects.

**Methods:**

This study included 16 patients who underwent 18 one‐stage rTKAs using custom‐made implants due to aseptic loosening between 2016 and 2023. Conventional revision systems failed to provide appropriate femoral or tibial fixation due to severe conical longitudinal bone defects classified as Anderson Orthopaedic Research Institute (AORI) type III. Clinical outcomes were assessed using the knee injury and osteoarthritis outcome score (KOOS), Oxford knee score (OKS), visual analogue scale (VAS) for pain, range of motion (ROM), walking time and procedure‐related complications. Implant costs were compared between customised and standard implants.

**Results:**

Mean follow‐up was 51 months (range, 24–100), with patients having an average of five previous surgeries (range, 2–8). During follow‐up, three patients (17%) reported complications: one periprosthetic fracture, one periprosthetic joint infection and one failure of the modular stem component. Mean KOOS improved from 31 to 80 (*p* < 0.001), OKS from 47–32 points (*p* < 0.001) and pain on the VAS decreased from 8.1 to 3.1 (*p* < 0.001).

**Conclusions:**

Customised implants for one‐stage rTKA present a promising cementless fit‐and‐fill fixation option for patients with severe longitudinal bone defects, particularly when standard knee revision implants, including cones and sleeves, are no longer suitable. Yet, these results are just midterm and small sample size‐based and therefore long‐term results in larger patient numbers need to be awaited before a final conclusion can be made.

**Level of Evidence:**

Level IV.

**Abbreviations**
AORI: Anderson Orthopaedic Research Institute
BMI: body mass index
CT: computer tomography
DAIR: debridement antibiotics implant retention
DFR: distal femoral replacement
DHS: dynamic hip screw
EBJIS: European Bone and Joint Infection Society
KM: Kaplan–Meier
KOOS: knee injury and osteoarthritis outcome score
MARS: metal artefact reduction sequences
OKS: Oxford knee score
PJI: periprosthetic joint infection
ROM: range of motion
rTKA: revision total knee arthroplasty
UCS: unified classification system
VAS: visual analogue scale

## INTROUCTION

Despite the growing number of revision surgeries, the survivorship of revision total knee arthroplasty (rTKA) remains lower compared to primary total knee arthroplasty (TKA). While the revision risk for primary TKA is estimated at 5% within 10 years postoperatively, the risk of re‐revision following rTKA ranges from 10% to 14% within 4–8 years postoperatively [4, 11, 16]. Additionally, the incidence of aseptic loosening rises with each subsequent revision [13]

The main reasons for re‐revision are periprosthetic joint infection (PJI) and aseptic loosening [12, 13, 21]. The latter seems to improve since sleeves and cones also offer an additional metaphyseal fixation option following the two‐zonal fixation concept of Morgan‐Jones emphasising the importance of metaphyseal reinforcement [20]. Recent meta‐analyses and long‐term series have shown that metaphyseal sleeves and cones achieve substantially lower re‐revision rates than: Fischer et al. reported 5.5% for sleeves versus 14.4% for cones at ≥5 years [7], Agarwal et al. observed 6.7% aseptic loosening at ≥7 years for sleeves [1], and De Martino et al. documented durable mid‐term fixation of tantalum cones with no loosening or migration at a mean 6‐year follow‐up [18]. These outcomes correlate with defect size (AORI grade) and bone quality, as highlighted in recent systematic reviews [22, 23]. However, in some revision cases, the metaphysis is too deficient to provide adequate fixation for standard sleeves and cones. In these situations, isolated diaphyseal fixation may be the only remaining option, although it carries a high risk of failure due to the transmission of shear forces primarily to the diaphyseal stem fixation, especially in hinged TKA designs [2, 6]. Furthermore, multiple previous debridements often reduce cancellous bone volume, making cemented stem fixation a less reliable option and increasing the risk of early aseptic loosening [3]. Cementless fit‐and‐fill stem fixation—addressing both the individual conical longitudinal defects of the diaphysis (which could potentially be classified as ‘Anderson Orthopaedic Research Institute [AORI] type 4’) and the natural femoral antecurvation—can therefore be considered as an alternative option. Recent advancements in medical technology have introduced custom‐made stem implants as a promising solution for these complex scenarios. Several studies have reported favourable outcomes of 3D‐printed titanium metaphyseal cones, enhancing mechanical stability and patient satisfaction, particularly in revision cases involving severe bone defects [5, 9, 17]. However, those studies are based on a two‐zonal fixation of rTKA.

Currently, there is a knowledge gap regarding the outcomes of individualised fit‐and‐fill implants that provide mono‐zonal diaphyseal fixation. This study aimed to evaluate the clinical outcomes and complication rates of customised femoral and tibial stem implants used in one‐stage aseptic rTKA for patients with severe longitudinal bone defects.

## MATERIALS AND METHODS

### Patients enrolment

**Figure 1:**
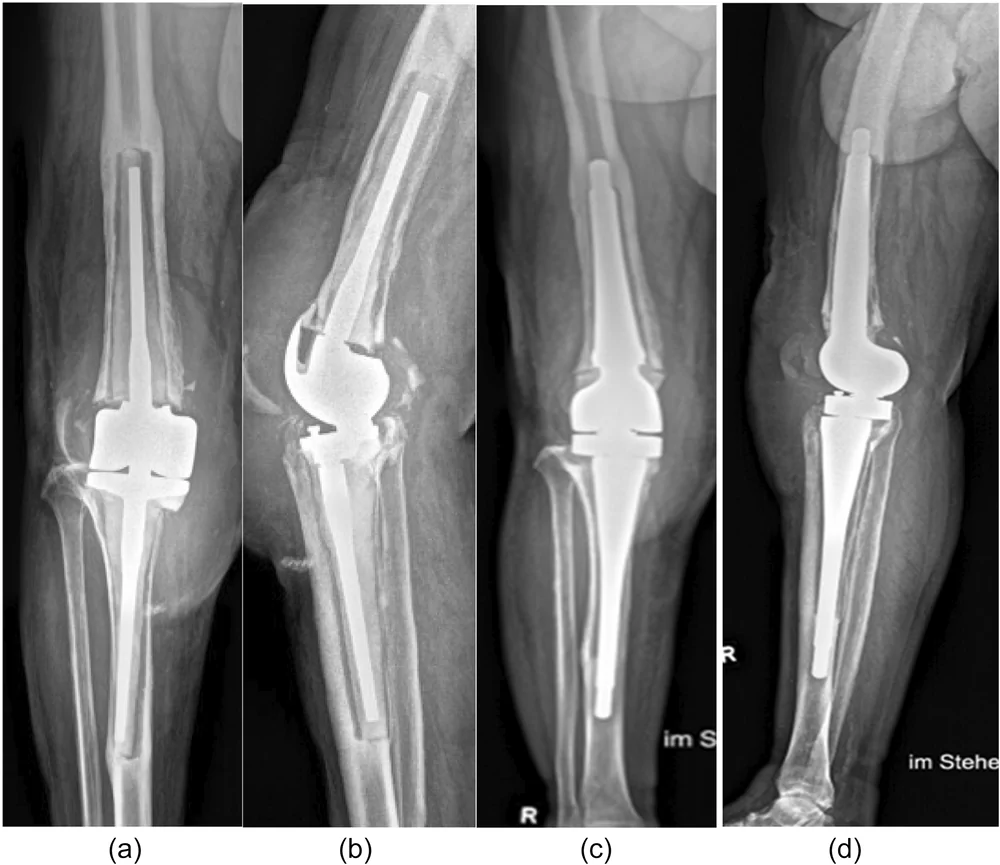
(a, b) Preoperative X‐rays (a.p.und lateral view) before one‐stage revision knee arthroplasty of the femur and tibia. (c, d) Postoperative X‐rays (a.p.und lateral view) after revision and implantation of both the femoral and tibial revision implants.

**Figure 2:**
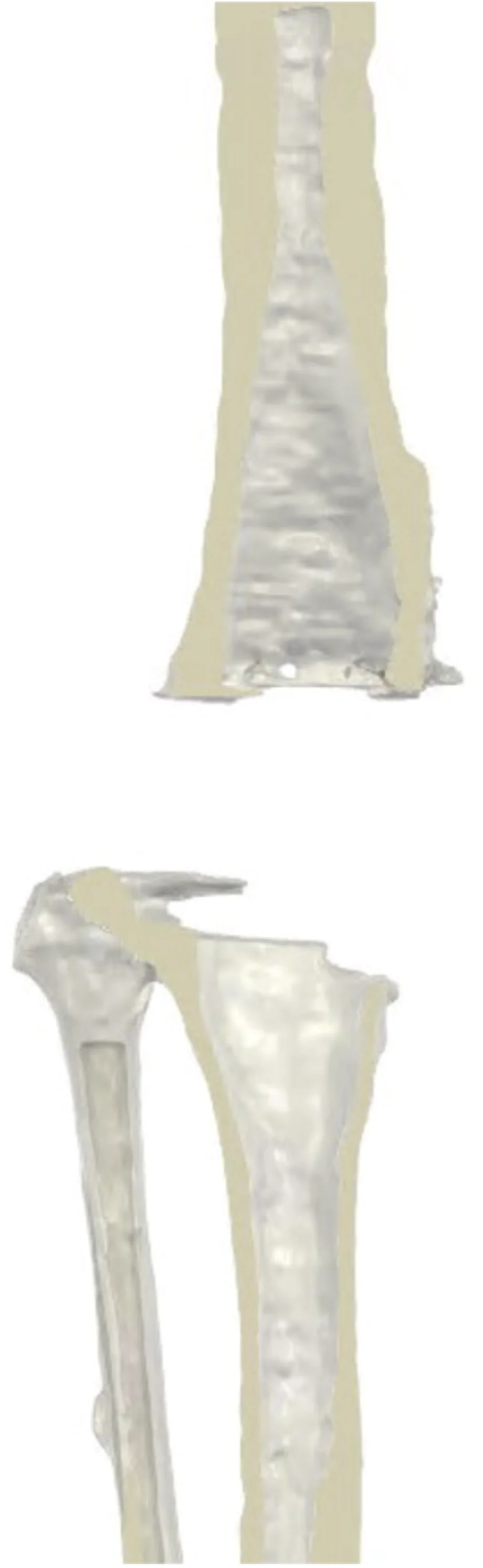
Preoperative computer tomography 3D‐reconstruction and sketch of femoral and tibial bone defect.

For this retrospective cohort study, all cases of customised aseptic revision knee arthroplasty (OptiStem^©^, Waldemar Link GmbH & Co. KG) who were admitted to our department between January 2016 and December 2023 were identified in the hospital internal information system and screened regarding inclusion and exclusion criteria. Inclusion criteria were aseptic loosening and complex asymmetric bone defects classified as AORI type III. Of note, only patients with severe AORI type III defects were considered for a customised stem solution. These defects were characterised by the absence of the epiphysis, a circumferentially compromised metaphysis, and pronounced metaphyseal–diaphyseal widening, most commonly resulting from multiple prior revision procedures for septic or aseptic loosening (Figures 1a,b and 2). The severe meta‐/diaphyseal defect—usually of at least 6 cm in length ‐ which could not be sufficiently addressed using off‐the‐shelf cortical cones or sleeves in combination with standard cemented or cementless stems. Usually, the nearly circumferentially compromised metaphysis allowed no proper use of sleeves or cones so the extent of bone loss typically allowed only a mono‐zonal fixation at the level of the enlarged diaphysis for the planned revision TKA. Due to the irregular enlarged diaphyseal geometry and, in femoral cases, the additional challenge of femoral antecurvature, standard cementless off‐the‐shelf stems were not feasible. While a cemented stem would technically represent an alternative, the diaphyseal bone in these multiply revised cases was usually sclerotic and lacked sufficient cancellous structure to ensure adequate cement interdigitation, thus raising concerns regarding early aseptic loosening. Therefore, in situations where neither standard cementless nor cemented stems were expected to provide reliable fixation, a customised cementless mono‐zonal stem was considered as a limb salvage option. This strategy was discussed in every case in detail with the patient, the healthcare provider and the implant manufacturer. If considered feasible, planning process began as described in the following section.

Aseptic condition of the revision was confirmed according to the European Bone and Joint Infection Society (EBJIS) criteria [19], including preoperative joint aspiration and intraoperative tissue samples for microbiological analysis. Patients without severe bony defects who received individualised rTKA due to an extraordinary under‐ or oversized implant (*n* = 2) were excluded from the study. None of the patients presented with functional extensor mechanism insufficiency or rupture prior to surgery. Demographic and clinical data were collected, including age at rTKA, gender, body mass index (BMI) and the number and aetiology (septic vs. aseptic) of previous knee arthroplasty procedures.

### Outcome parameters

Outcome parameters, including the knee injury and osteoarthritis outcome score (KOOS) and Oxford knee score (OKS, best = 12, worst = 60), and pain on visual analogue scale (VAS), were assessed preoperatively and at 6 weeks, 3 months and annually during standard follow‐up visits. For additional subjective evaluation, patients were asked at the latest follow‐up whether they would have preferred amputation over rTKA. Clinical outcome was evaluated by range of motion (ROM) and maximum walking time (min) preoperatively and annually. Radiographic assessment for signs of loosening was performed using standardised postoperative anteroposterior, lateral and full‐length radiographs. All postoperative complications, including PJI, aseptic loosening, periprosthetic fractures, instability and wound healing disorders (haematoma, delayed wound healing) were documented. The number and type of subsequent revision procedures, including irrigation and debridement, liner exchange, or component revision, were recorded. Implant costs of custom‐made implants were assessed and compared to standard knee revision implants. The costs of standard implants (combined stem and cone) were €1995 (femoral) and €1495 (tibial).

### Planning and manufacturing process

**Figure 3:**
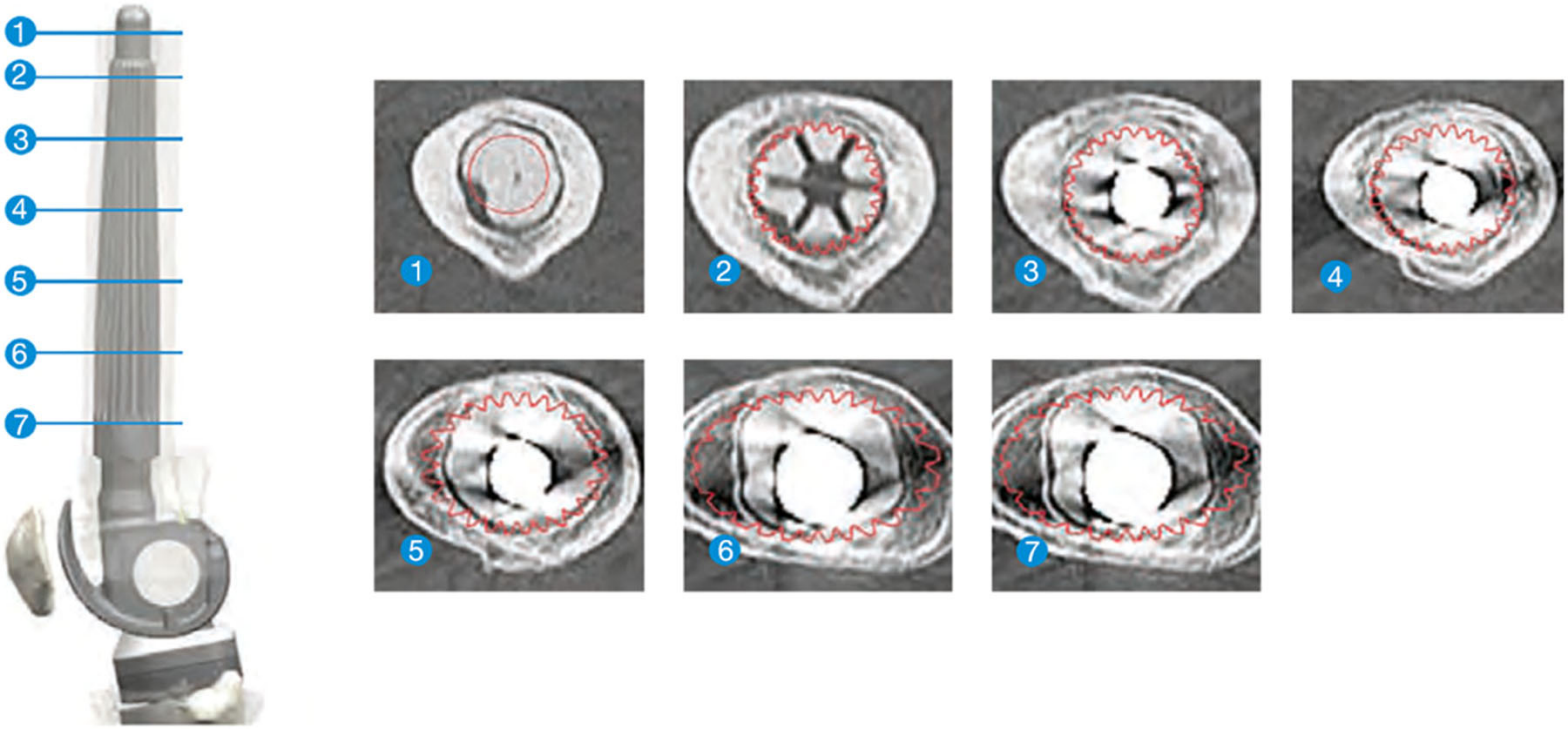
Radiological planning of the femoral implant based on CT scans including customised conical shape and antecurvation. The subfigures (1–7) on the right side demonstrate the axial view of the femur from the corresponding markings on the left side. CT, computer tomography.

**Figure 4:**
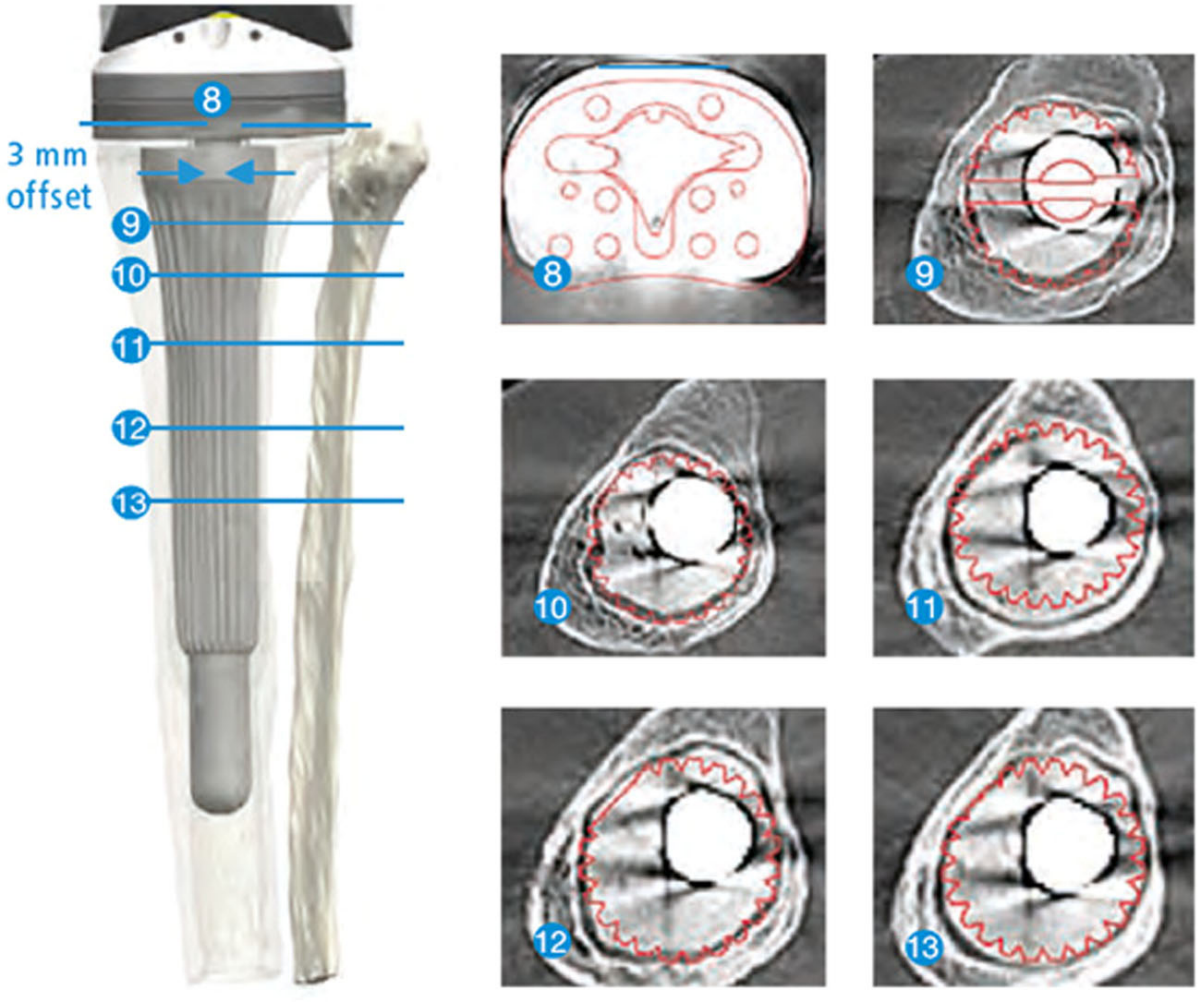
Radiological planning of the tibial implant based on CT scans including customised offset and stem alignment. The subfigures (8–13) on the right side demonstrate the axial view of the tibia from the corresponding markings on the left side. CT, computer tomography.

The base components of the knee system were the Link^©^ Endo‐Modell rotating hinge system and the Megasystem‐C distal femoral replacement (DFR) (Waldemar Link GmbH & Co. KG). The planning and manufacturing of each implant were based on a standardised computer tomography (CT) scan protocol using metal artefact reduction sequences (MARS) (Figures 3 and 4). Dicom data were provided to manufacturers engineering department and close exchange between surgeons and engineer formed first draft concerning surgical approach, new implant size and stem length. For planning of the customised fill‐and‐fit stem the bone defect, targeted for implant revision, was divided into several segments. At each level, the extent of the bone defects and the shape of the planned stem designed to fill these defects were analysed. The optimal implant design was determined through close and repetitive cooperation between the manufacturer and the surgical team.

**Figure 5:**
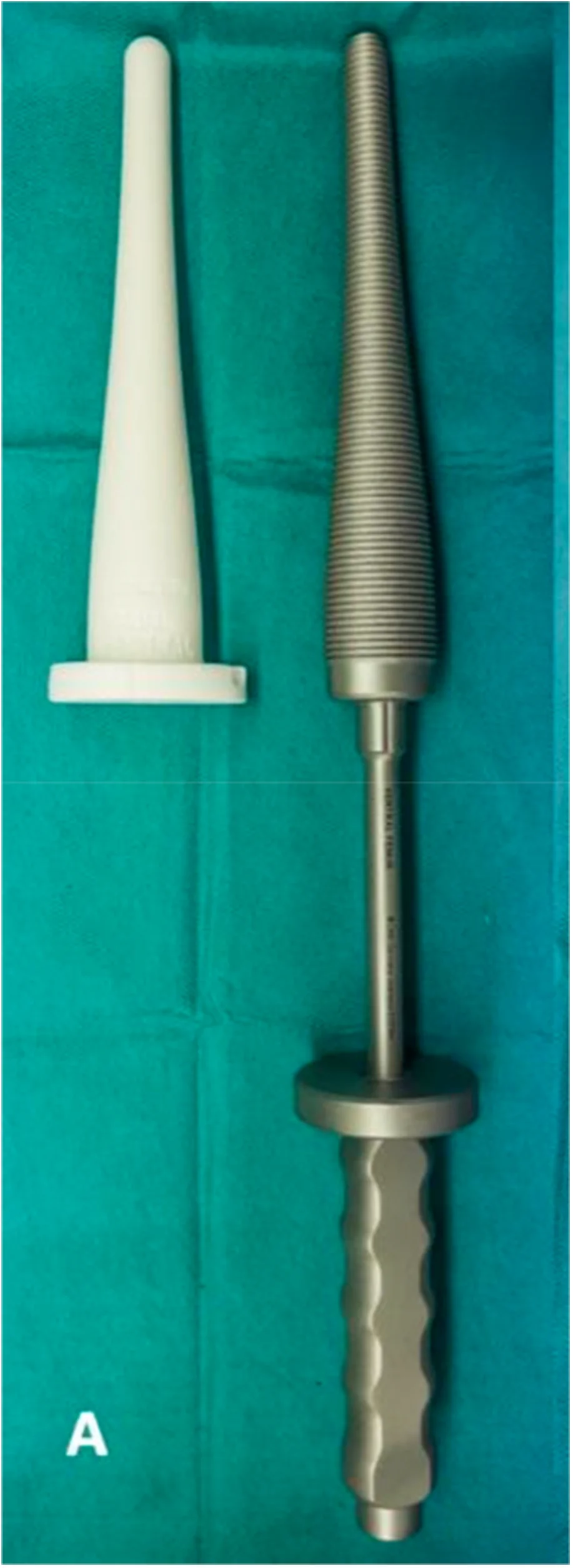
A special rasp impactor (right) is supplied with each customised implant. The fit of the implant can be tested with a 3D dummy (left).

Individual implants, based on the standard oval‐shaped OptiStems design, were manufactured from forged titanium. Wrought titanium alloy (Ti6V4‐Tilastan‐S) rod material was used to machine the individual personalised stem as a cementless version with Wagner‐style grooves and ribs. Trial implants, special rasps and bone models using 3D‐printing were produced from various biocompatible materials, most commonly polyamide (Figure 5).

### Statistical analysis

Statistical analysis was performed using SPSS Version 24 (IBM Corp.). Descriptive statistics for clinical data, including mean, standard deviation, minimum and maximum values for continuous variables, were calculated. Differences in patient outcome parameters (OKS, KOOS and VAS), and clinical outcome parameters (ROM, walking distance) were assessed using the Wilcoxon test for non‐normally distributed data and paired *t*‐tests for normally distributed data.

## RESULTS

**Figure 6:**
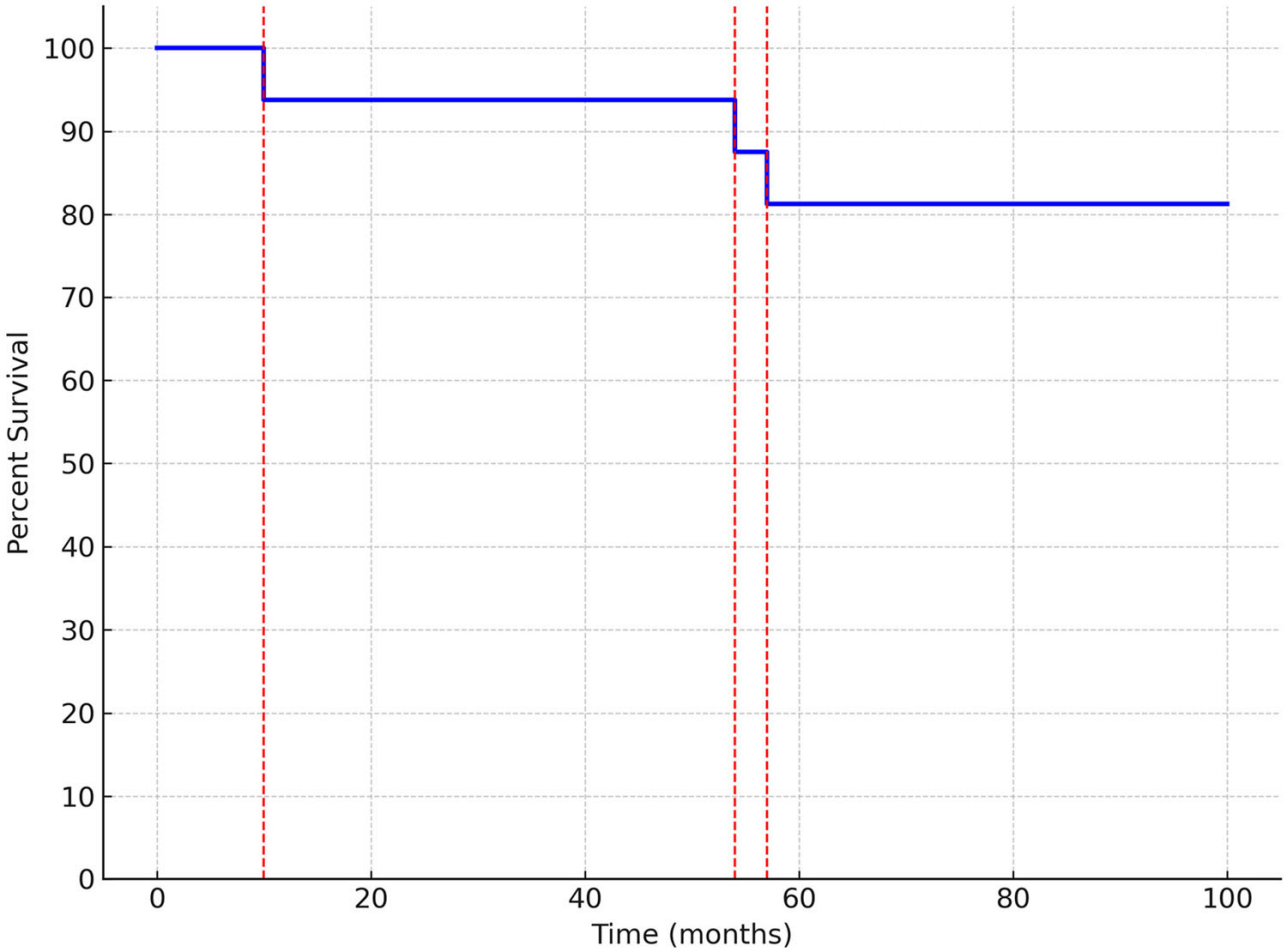
Kaplan–Meier implant suvivorship curve for survival free of any revision.

Sixteen consecutive patients (nine women, seven men) received individualised custom‐made knee revision stem implants. Mean follow‐up was 51 months (range, 24–100), with patients having undergone an average of five previous surgeries (range, 2–8). Survivorship is shown in Figure 6.

The mean age at the time of surgery was 67 years (range, 36–80), and the mean body mass index (BMI) was 28.5 kg/m^2^ (range, 19.9–35.6). Demographic data and implant costs per case are presented in Table 1.

**Table 1:** Patient demographics and cost of implants.

	Case ID	Patient ID	Age (years)	Sex	BMI (kg/m^2^)	N Pre‐Surg	FU	H/O PJI	Localisation of implant	Cost of implants (Euro)	Comment
	1	1	36	M	25	2	100	No	Femur	2382	
	2	2	72	F	21.5	7	80	Yes	Femur	11,186	
	3	3	69	F	21.8	3	68	Yes	Tibia	5562	Died 06/2023
	4	4	72	F	37.0	7	65	Yes	Tibia	12,119	
	5	5	55	F	28.2	2	60	No	Femur	4602	
	6	6	64	M	35.2	4	55	Yes	Femur	14343	
	7	7	67	M	31.5	4	48	No	Femur	9064	
	8	8	80	F	28.0	2	44	No	Femur	4627	
	9	9	73	M	35.6	9	48	Yes	Femur and Tibia	9216 (both)	
	10	10	66	M	29.7	8	48	Yes	Femur	4946	
	11	11	60	F	19.9	3	47	Yes	Femur	4729	
	12	12	73	M	29.0	5	45	Yes	Femur	4851	
	13	13	80	F	31.9	5	45	Yes	Femur and Tibia	8350 (both)	
	14	14	78	F	27.7	5	40	Yes	Femur	4887	
	15	6	66	M	27.5	6	30	Yes	Tibia	3895	
	16	15	73	M	33.7	6	36	Yes	Femur	8243	
	17	16	56	F	17.6	8	31	Yes	Tibia	4486	
	18	7	70	M	31.5	5	24	No	Tibia	5600	
Mean			67		28.5	5.1	51				

Abbreviations: BMI, body mass index; FU, follow‐up; H/O, history of; N Pre‐Surg, number of prior surgeries including primary implantation; PJI, periprosthetic joint infection.

Ten patients received customised distal femoral implants, four patients received tibial implants, and two patients received both femoral and tibial revision in a single operation (Figure 1c,d).

### Clinical and radiological outcome

**Figure 7:**
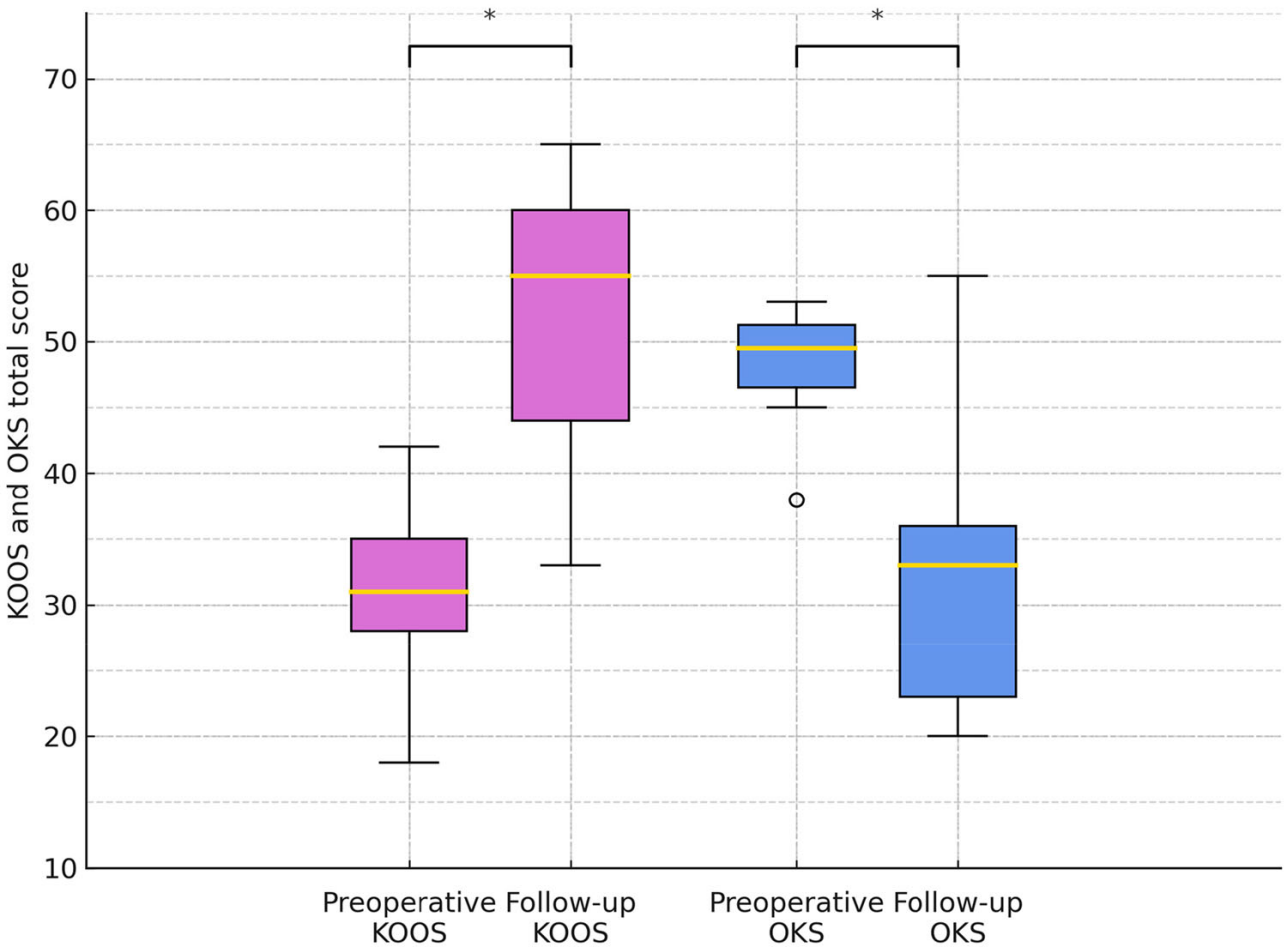
Patient‐reported outcome measures: KOOS and OKS preoperative and at latest Follow‐up. *p < 0.001**.** KOOS, knee injury and osteoarthritis outcome score; OKS, Oxford knee score.

The mean KOOS improved from a preoperative mean of 30.7 ± 5.7 to a postoperative mean of 50.6 ± 9.8 (*p* < 0.001). Similarly, the mean OKS decreased—indicating improvement—from 46.7 ± 5.3 to 31.5 ± 9.3 (*p* < 0.001) (Figure 7). Mean preoperative pain on VAS was 8.1 ± 1.2 and decreased to 3.1 ± 1.6 postoperatively (*p* < 0.001).

Range of motion did not change significantly, with a mean ROM of 98° ± 12.1 preoperatively and 98° ± 13.7 postoperatively (*p* = 0.77). Walking time significantly increased from a preoperative mean of 6.8 min ± 4.9–20.1 min ± 16.4 postoperatively (*p* < 0.05).

All patients were asked whether they would have preferred amputation over implant revision. No patient retrospectively favoured amputation.

All customised implants, except for one, fit the shape of the complex bone defect. In one case, the femoral defect differed slightly from the preoperative plan, likely due to progressive loosening after CT acquisition, which resulted in a deeper diaphyseal seating of the stem and required the use of a compatible off‐the‐shelf lengthening segment. In summary, no implant had to be discarded. Intraoperatively, no periprosthetic fractures were observed. Radiographic analysis showed no cases of aseptic loosening requiring revision. In one patient, a 3 mm subsidence of tibial customised implant was noted on radiographs 3 months post‐operatively, with no need for revision. In all other patients, no significant radiolucent lines or implant position deviation were observed.

Survival free of any revision was estimated using the Kaplan–Meier (KM) method (Figure 6).

### Complications

Overall revision rate was 17% (*n* = 3). No intraoperative complication occurred. Postoperatively, one fracture of the femoral neck (Unified Classification System [UCS] type C) occurred proximal to the customised distal femoral replacement after adequate trauma, 10 months after surgery. The patient was undergoing osteosynthesis using a dynamic hip screw (DHS) and initially recovered well. However, 54 months after DHS fixation, a subsequent periprosthetic fracture occurred proximal to the femoral stem implant, resulting in a push‐through‐stem. One patient required revision surgery due to failure of the modular implant connection between the femoral stem to articular component, occurring 10 months postoperatively after significant trauma slipping and falling to the floor. Another patient was diagnosed with hematogenous PJI 57 months after the operation, which was successfully treated with a debridement, antibiotics and implant retention (DAIR) procedure.

### Cost of implants and manufacturing

The mean cost of customised femoral and tibial stem implants, including form‐fit compressor and trial, was €6154 (range, €2382 to €14,383), representing a 310% increase over standard femoral implants and 430% increase over standard tibial implants. A detailed overview is provided in Table 1.

## DISCUSSION

This study demonstrates that customised, mono‐zonal diaphyseal fit‐and‐fill cementless stem implants for the femur and tibia offer a promising limb‐salvage solution, achieving satisfactory mid‐term outcomes in complex rTKA cases with severe bone defects. Despite the expected functional limitations associated with multiple rTKAs, patients reported significant improvements in both function and pain. At the final follow‐up, none of the patients retrospectively expressed a preference for amputation, which can be considered a satisfactory outcome for this complex cohort.

So far, only one study with a short‐term follow‐up of 24 months in 10 cases has evaluated radiological outcomes and implant survivorship of customised meta‐ and diaphyseal‐fixating implants in periprosthetic joint infection [24]. Notably, only four cases received an isolated diaphyseal customised stem fixation of the femur. All other cases received individualised cones, indicating that two‐zonal fixation was possible in most cases, and therefore comparability to the results of the present study may be limited.

The observed overall revision rate of 17% at a mean follow‐up of 51 months appears favourable when compared to rates reported in the literature for “standard” megaprostheses. Fraser et al. reported a revision‐free survival of 58% at 8 years in a cohort of 247 cases treated with a rotating‐hinge megaprosthesis [8]. Höll et al. observed a mid‐term revision rate of 55% after a mean follow‐up of 34 months (range, 10–84 months) [10]. Smith et al. reported a complication rate of 34% 2 years after septic and aseptic revision with megaprosthesis [25].

In addition to PJI, aseptic loosening remains a major complication in multiple rTKAs [13, 14]. Multiple rTKAs lead to a combination of reduced bone quality and the occurrence of large bony defects, resulting in impaired two‐zonal fixation options as well as increased shearing forces in highly constrained implants, which favour the occurrence of early aseptic loosening [2, 3, 12, 14]. A previous study reported an average rTKA implant survivorship of only 24 months in cases with four to five prior surgeries [13]. In contrast, this present study demonstrated a longer average implant survival of 51 months, with no cases of aseptic loosening. These results suggest that cementless mono‐zonal diaphyseal fit‐and‐fill stem implants may be a promising option in cases in which cones and sleeves are no longer an option due to a major deficient metaphysis. Nevertheless, it should be acknowledged that exclusive diaphyseal fixation imposes substantial biomechanical stress on the modular junction between the stem and the articular component, as evidenced by the single case of junction failure observed in this cohort.

Another potential risk factor for failure is the interval between the diagnosis of aseptic loosening, the indication for a customised implant, and the final surgical procedure. This period depends on case complexity, approval of additional costs by the healthcare provider, and the manufacturing process, and may extend up to 4 months. Prolonged waiting times increase the risk of progressive bone loss relative to the planning CT, potentially resulting in a suboptimal fit of the customised stem like faced in one case in the recent study.

An alternative approach to individualised femoral fit‐and‐fill fixation has been described by Li et al. [15]. A key technical difference is that a complete 3D‐adaption to the individual anatomy was not utilised; instead, only the antecurvation angle and the intramedullary diameters at different reference points were considered, resulting in less contact area compared to the complete 3D‐based print used in the present study. The examined cohort, consisting predominantly of oncology patients, showed no cases of aseptic loosening or other revision‐related failures at a mean follow‐up of 80 months. However, as the majority of patients were oncological cases undergoing level resections, they do not represent the typical multiple revision TKA population described in the AORI system. Furthermore, the bone quality of the remaining diaphysis in these patients is typically superior to that of patients who have undergone multiple surgical debridements. Despite the limited comparability between these studies, a highly individualised cementless fit‐and‐fill approach appears to be key for successful mono‐zonal diaphyseal fixation.

Although customised implants are associated with 3.1‐fold (femoral) to 4.3‐fold (tibial) higher initial costs (mean €6154; range: €2382–€14,383), these must be viewed in the context of the complex clinical scenarios in which they are used. In this cohort, all patients presented with severe AORI type III defects affecting the diaphysis, where standard implants failed to provide sufficient two‐zonal fixation, rendering individualised solutions the only viable alternative to amputation.

The present study has several limitations. First, it is a retrospective case series with a small number of patients. However, it represents the largest and most homogenous cohort in the current literature, as it includes only patients with customised, mono‐zonal diaphyseal fit‐and‐fill cementless stem implants of the femur and tibia. In all cases, additional metaphyseal fixation was no longer feasible. A second limitation is the lack of a control group, which is inherent to the retrospective design of the study. As the cohort exclusively included patients for whom standard rTKA was no longer viable due to extensive metaphyseal and diaphyseal bone loss, leaving amputation as the only alternative to customised implants, the inclusion of a control group was ethically unjustifiable.

## CONCLUSION

Customised cementless fit‐and‐fill stems offer a promising, limb‐preserving salvage option in rTKA with limited but satisfactory clinical and radiographic mid‐term outcomes in cases of complex longitudinal bone defects. Despite providing only mono‐zonal fixation of those implants, no case of aseptic loosening were observed. Long‐term data from larger patient series are needed before definitive conclusions on the use of these customised cementless implants can be drawn.

## AUTHOR CONTRIBUTIONS

**Stefanie Donner**: Conceptualisation; methodology; data curation; formal analysis; investigation; methodology; project administration; visualisation; writing—original draft; writing—review and editing; approval of the final manuscript. **Clemens Gwinner**: Investigation; supervision; writing—review and editing. **Henryk Haffer**: Investigation; writing—review and editing. **Carsten Perka**: Conceptualisation; formal analysis; investigation; methodology; supervision; writing—review and editing, approval of the final manuscript. **Stephanie Kirschbaum**: Data curation; formal analysis; investigation; methodology; project administration; supervision; validation; writing—review and editing; approval of the final manuscript.

## CONFLICT OF INTEREST STATEMENT

Carsten Perka reports relationships with J&J Medtech, Zimmer Biomet, Smith and Nephew, outside and unrelated to the submitted work. Stefanie Donner reports relationships with Smith and Nephew, outside and unrelated to the submitted work.

## ETHICS STATEMENT

This study was approved by the Institutional Review Board of Charité ‐ Universitätsmedizin Berlin, Registry Number: (EA4/109/23). All patients provided written informed consent for the use of their anonymized clinical and radiological data for research and publication purposes.

## Data Availability

The data that support the findings of this study are available from the corresponding author upon reasonable request.
